# Response of N_2_O emissions to biochar amendment in a cultivated sandy loam soil during freeze-thaw cycles

**DOI:** 10.1038/srep35411

**Published:** 2016-10-17

**Authors:** Xiang Liu, Quan Wang, Zhiming Qi, Jiangang Han, Lanhai Li

**Affiliations:** 1 State Key Laboratory of Desert and Oasis Ecology, Xinjiang Institute of Ecology and Geography, Chinese Academy of Sciences, Urumqi, 830011, Xinjiang, China; 2 University of Chinese Academy of Sciences, Beijing, 100049, China; 3 Faculty of Agriculture, Shizuoka University, Shizuoka 422-8529, Japan; 4 College of Biology and the Environment, Nanjing Forestry University, Nanjing, 210037, China

## Abstract

In the last decade, an increasing number of studies have reported that soil nitrous oxide (N_2_O) emissions can be reduced by adding biochar. However, the effect of biochar amendment on soil N_2_O emissions during freeze-thaw cycle (FTC) is still unknown. In this laboratory study, biochar (0%, 2% and 4%, *w/w*) was added into a cultivated sandy loam soil and then treated with 15 times of FTC (each FTC consisted of freeze at −5/−10 °C for 24 h and thaw at 5/10 °C for 24 h), to test whether biochar can mitigate soil N_2_O emissions during FTC, and estimate the relationships between N_2_O emissions and soil inorganic nitrogen contents/microbial biomass content/enzyme activities. The results showed that biochar amendment suppressed soil N_2_O emissions by 19.9–69.9% as compared to soils without biochar amendment during FTC. However, N_2_O emissions were only significantly correlated to soil nitrate nitrogen (NO_3_^−^-N) contents, which decreased after biochar amendment, indicating that the decreased soil nitrification by adding biochar played an important role in mitigating N_2_O emissions during FTC. Further studies are needed to estimate the effectiveness of biochar amendment on reducing freeze-thaw induced N_2_O emissions from different soils under field conditions.

The potent greenhouse gas nitrous oxide (N_2_O) plays an important role in the global biogeochemical nitrogen (N) cycle, affecting both ongoing global warming and stratospheric ozone depletion^1^,^2^. Its atmospheric concentration has increased dramatically from approximately 270 ppbv in the pre-industrial era to 322.5 ppbv in 2009^1^. Among the sources of atmospheric N_2_O, agricultural soils have been identified as the most important one, which accounts for approximately 60% of the global anthropogenic N_2_O emissions^1^,^3^. The loss of N from soils will also decrease N availability to crops and affect crop productivity negatively^4^. Hence, new agricultural management practices are needed to reduce N_2_O emission as well as maintain N availability of soils.

Biochar, which is produced from the slow pyrolysis (<700 °C) of biomass with partial or complete exclusion of oxygen, has a relatively high carbon (C) content, surface area, and cation exchange capacity compared to unheated biomass^5^. In the last decade, an increasing number of studies have suggested that adding biochar into soils may have the ability to reduce soil N_2_O emissions^6^. However, these impacts vary across soil and biochar types and also strongly depend on soil conditions^6^. For instance, Bruun *et al*.^7^ and Clough *et al*.^8^ found that the combined applications of biochar and anaerobically digested slurry or bovine urine could increase soil N_2_O emissions; while in a subtropical pasture where precipitation is high in summer, Scheer *et al*.^9^ reported that biochar amendment had no effect on soil N_2_O emissions. As a result, the response of soil N_2_O emissions to biochar amendment may exhibit various behaviors under different soil conditions.

Soil freeze-thaw cycle (FTC), which is caused by the phase transition of soil water, is a common process during the non-growing season in mid-high latitude regions^10^. Enhanced soil N_2_O emissions during FTC have been reported under both field and laboratory conditions^11^,^12^,^13^. The mechanisms responsible for the burst of N_2_O emissions after thawing have been widely discussed. Enhanced microbial metabolism by the accumulated substrates during thaw periods is considered as the most likely reason^14^, since several studies indicated that FTC might induce significant increases in soil N mineralization^15^,^16^. A recent study by Case *et al*.^17^ showed that adding biochar into soils could also stimulate soil N mineralization and nitrification, while suppressed cumulative production of N_2_O by 91%. Therefore, the relationship between soil inorganic N contents and N_2_O emissions may be complicated after biochar amendment during FTC.

Aside from the availability of substrates, soil microbial biomass and enzyme are two other important factors that influence soil N_2_O emissions because they involve in catalytic reactions and nutrient mineralization^18^,^19^. For instance, Wick *et al*.^20^ reported that soil N_2_O emissions were positively correlated to soil microbial biomass N (SMBN) and β-glucosidase activities during a dry season; Bai *et al*.^21^ found that soil urease activity was an indicator of N_2_O emission because of the close relationship between urease activity and nitrification. Previous studies have demonstrated that soil microbial biomass and enzyme activities can be changed by adding biochar^22^,^23^,^24^. However, the observation periods of these studies all focused on the growing season. During FTC, the dynamics of soil microbial biomass and enzyme activities as well as their relationships with soil N_2_O emission after adding biochar are poorly understood.

Soils in mid-high latitude regions are projected to experience higher frequencies and larger amplitudes of FTC in the context of climate change, which in turn release more N_2_O into the atmosphere^25^. Although biochar amendment is a potential amendment to mitigate soil N_2_O emissions, very limited information is available on the effect of biochar amendment on soil N_2_O emission during FTC. The objectives of this laboratory study were to: (1) investigate the effects of biochar amendment on soil N_2_O emission, inorganic N contents, microbial biomass and enzyme activities during FTC; (2) estimate the relationships between N_2_O emissions and soil inorganic N contents/soil microbial biomass contents/enzyme activities under the joint effects of FTC and biochar amendment. More specifically, we tested the hypothesis that the N_2_O bursts during FTC will be suppressed by biochar amendment.

## Materials and Methods

### Soil collection and analysis

In May 2015, soil samples (0–20 cm) were collected from a farmland (43°27′ N, 82°54′ E) cultivated with corn in the Ili River Valley, Xinjiang Uygur Autonomous Region, northwest China. The surface soils in this area usually experience seasonal freeze-thaw process during early spring. The soil was classified as Typic Haploboroll (USDA) with a sandy loam texture (4.2%, 23.2% and 72.6% for clay, silt and sand, respectively). Collected soil samples were air-dried, homogenized and grounded to pass through a 2 mm nylon fiber sieve before experimental use.

Soil organic C (SOC) was measured using the H_2_SO_4_-K_2_Cr_2_O_7_ oxidation method, while soil total N (TN) was detected using an automatic azotometer (Kjeltec 8400, FOSS, Denmark) according to the Kjeldahl method. Soil ammonium N (NH_4_^+^-N) and nitrate N (NO_3_^−^-N) were determined using a continuous flow analyzer (AA3, SEAL Analytical, Germany) with 0.01 M CaCl_2_ extracts (1:10, *w/v*)^26^. Soil pH and electric conductivity were measured in a volume ratio (H_2_O) of 1:5 (*w/v*) using a pH meter (SevenEasy, Mettler-Toledo, Switzerland) and an electric conductivity meter (DDSJ-308A, Rex, China), respectively. Soil texture was analyzed using a laser diffraction particle analyzer (Mastersizer 2000, Malvern, UK). SMBN was measured using the chloroform fumigation–K_2_SO_4_ extraction method (1:4, *w/v*). The extracts were analyzed at 280 nm using an UV spectrophotometer (Cary 60, Agilent Technologies, USA)^27^. Activities of urease and protease were determined using the indigo colorimetric method and the ninhydrin colorimetric method with urea and casein as substrates, respectively^28^,^29^. Urease and protease activities were expressed as μg NH_4_-N g^−1^ h^−1^ and μg Tyr g^−1^ h^−1^, respectively. The physicochemical properties of soil are shown in Table 1.

### Biochar analysis

Biochar used for the experiment was made by the Seek Bio-Technology Company, Shanghai, China. It was produced using bamboo under a pyrolysis of 500–600 °C. The biochar was grounded to pass through a 2 mm nylon fiber and mixed thoroughly before experimental use.

**Figure 1:**
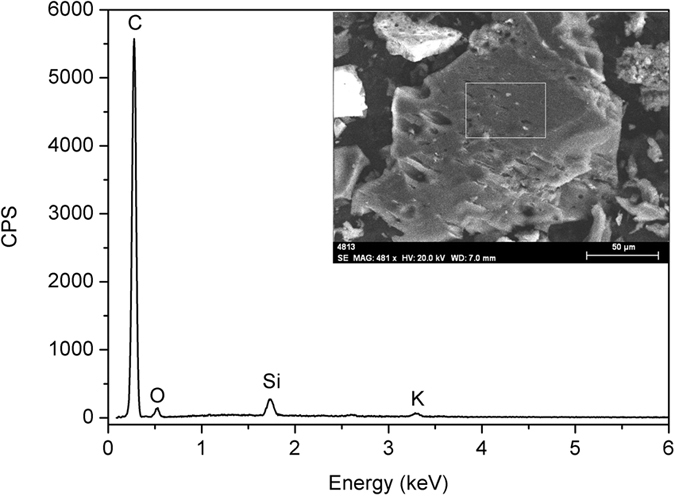
Scanning electron microscope image with energy dispersive X-ray spectra showing elemental composition of biochar.

Surface structure and elemental analysis of biochar were examined using a scanning electron microscope (Super 55VP, Zesis, Germany) associated with an energy dispersive X-ray spectroscopy (XFlash 5010, Bruker, Germany) (Fig. 1). The pH, electric conductivity, NH_4_^+^-N and NO_3_^−^-N of biochar were determined using the previously mentioned methods. To estimate ash content, 1.0 g of biochar was heated in a muffle furnace (LC-502, Koyo, Japan) at 500 °C for 8 h. The ash content was calculated from: ash content (%) = mass of ash/mass of biochar × 100^30^. The elemental (C, H, N and S) contents of biochar were measured using an elemental analyzer (vario MICRO cube, Elementar, Germany). The O content of biochar was determined by calculating the difference between 100% and the sum contents of ash, C, H, N and S. The physicochemical properties of biochar are also shown in Table 1.

### Experimental design

In 250 mL Erlenmeyer flasks, 60.0 g (oven-dry basis) of soils were mixed with 0% (BC0), 2% (BC2) and 4% (BC4) (*w/w*) of biochar, and then wetted with deionized water to reach 60% of water holding capacity. Each flask was covered with parafilms with several small holes to allow gaseous exchange and reduce the loss of soil water. All flasks were pre-incubated at 25 °C in dark condition for seven days. After that, flasks of each application rate were separated into two equal groups to experience different FTC treatments. Taking the field surface temperatures during spring freeze-thaw periods into consideration^31^, we set up two experiments with one in a small amplitude (−5 °C to 5 °C) of FTC (SFT) and the other in a large amplitude (−10 °C to 10 °C) of FTC (LFT). There were fifteen times of FTC in total and each included freeze at −5 or −10 °C for 24 h and thaw at 5 or 10 °C for 24 h. Six treatments in the present study were established and after the 1st, 3rd, 5th, 10th and 15th FTC, triplicate flasks of each treatment were randomly selected and destructively sampled. Soils were used for determining NO_3_^−^-N, NH_4_^+^-N and SMBN contents together with urease and protease activities. Furthermore, deionized water was added into each flask at the end of every two FTCs to compensate for the lost soil water. Table 2 shows the details of treatment layout and properties of the mixtures after pre-incubation.

### Gas sampling and analysis

Triplicate flasks for each treatment were sealed with rubber stoppers to collect gas samples at the end of 1st, 3rd, 5th, 10th and 15th FTC. In the middle of the stopper, a small hole was made and a plastic tube (0.2 cm in inner diameter, 10 cm in length) connected to a three-way stopcock was inserted into the hole. The gaps between stopper and tube were sealed with glue. The three-way stopcock was closed to make a gas-tight environment after covering. During a half hour closure period, a gas sample of approximately 2.5 mL was withdrawn using a gas-tight syringe at 0, 10, 20 and 30 min, respectively. The concentrations of N_2_O were detected within 3 days using a gas chromatograph (7890B, Agilent Technologies, USA), which was equipped with an electron capture detector. The carrier gas for N_2_O analysis was high-purity N_2_. N_2_O emissions were calculated using formula (1)^32^:


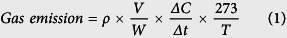


where *ρ* is the density of N_2_O at 0 °C (1.963 g m^−3^), *V* (m^3^) and *W* (kg) are the head space volume of the flask and the soil weight, respectively, Δ*C* is the change in N_2_O concentrations during the measurement period Δ*t* (h), and *T* is the absolute temperature. Cumulative emissions during the whole incubation were directly computed from the measured emissions and estimated by linear interpolation for days when no measurements were made.

### Statistical analysis

Three-way ANOVA was used to examine the differences in soil N_2_O emissions, NO_3_^−^-N, NH_4_^+^-N, SMBN contents, urease and protease activities among FTC amplitudes, biochar addition rates and FTC frequencies. Differences in cumulative N_2_O emissions between FTC amplitudes were tested using independent-samples t test, while differences in cumulative N_2_O emissions among biochar addition rates were examined using one-way ANOVA. Data sets have gone through the normality and heterogeneity tests and were converted to log-transformation (base 10) when the variances were unequal before analyses. Pearson correlation was employed to examine the correlations among N_2_O emissions, NO_3_^−^-N, NH_4_^+^-N, SMBN contents as well as urease and protease activities. Differences and correlations were considered statistically significant if *P* < 0.05 and highly significant if *P* < 0.01.

## Results

### NO_3_^−^-N, NH_4_^+^-N and SMBN contents

**Figure 2:**
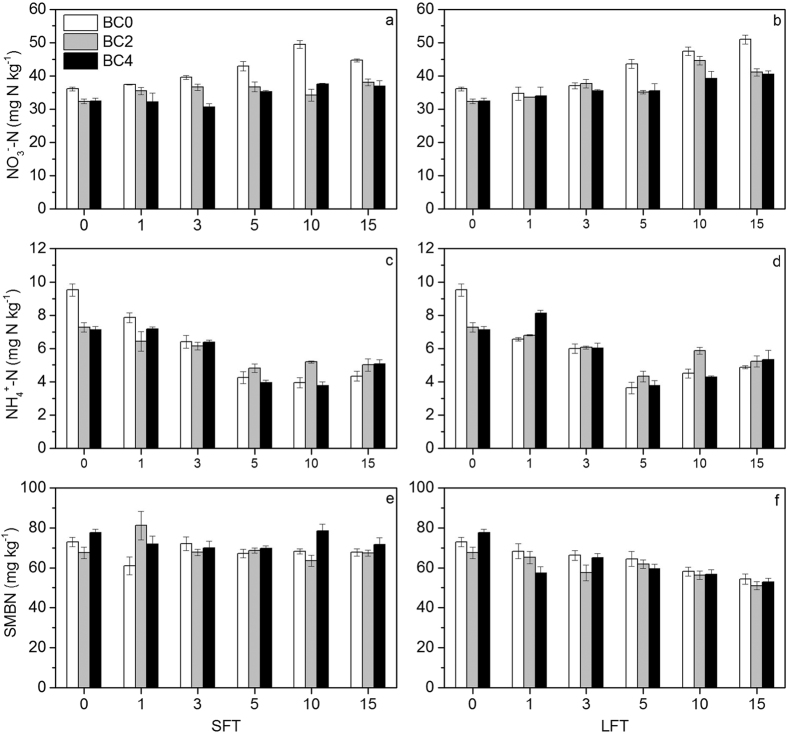
Changes in soil NO_3_^−^-N (**a**,**b**) and NH_4_^+^-N (**c**,**d**) and SMBN (**e**,**f**) contents by biochar additions during FTC ((**a**,**c**,**e**), FTC with small amplitude; (**b**, **d**, **f**), FTC with large amplitude). Bars represent the standard error of the mean (n = 3).

As shown in Fig. 2, soil NO_3_^−^-N contents of each treatment all increased during the incubation. As compared to the contents after pre-incubation, soil NO_3_^−^-N contents increased by 13.6–23.7% and 24.7–41.0% after 15 times of FTC under SFT and LFT, respectively. The largest increase was detected in LFT-BC0. Results of ANOVA analysis showed that soil NO_3_^−^-N content was significantly affected by FTC amplitude, biochar addition rate as well as FTC frequency (Table 3). Soil NH_4_^+^-N contents showed decreasing trends for all treatments in the first five FTCs and then slowly increased throughout the rest of FTCs (Fig. 2c,d). After the incubation, NH_4_^+^-N contents decreased by 29.1–54.6% and 25.4–49.0% under SFT and LFT compared with the contents after the pre-incubation, respectively. The effects of FTC amplitude and biochar addition rate on soil NH_4_^+^-N content were not significant when ignoring their interaction effects with FTC frequency (Table 3).

FTC amplitude and frequency had significant impacts on SMBN content (Table 3). As compared to the contents after the pre-incubation, 15 times of FTC decreased SMBN contents by 0.1–7.7% under SFT, while the range of SMBN content was only 61.2 to 81.3 mg kg^−1^ during the whole incubation. By contrast, LFT showed stronger effects on decreasing SMBN contents than SFT. SMBN contents of all treatments under LFT generally decreased with the increase of FTC times (Fig. 2e,f). After the 15th FTC, SMBN contents of LFT-BC0, LFT-BC2 and LFT-BC4 decreased to 54.5, 51.9 and 53.1 mg kg^−1^, respectively, which were the minimums of each treatment. However, biochar addition rate did not show a significant effect on SMBN content when ignoring its interaction effects with FTC amplitude and frequency (Table 3).

### Enzyme activities

**Figure 3:**
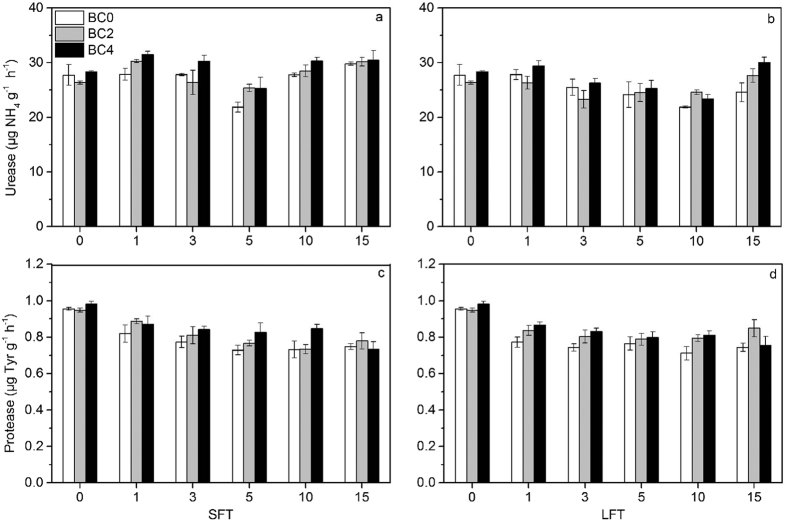
Changes in soil urease (**a**,**b**) and protease (**c**,**d**) activities by biochar additions during FTC ((**a**,**c**), FTC with small amplitude; (**b**,**d**), FTC with large amplitude). Bars represent the standard error of the mean (n = 3).

Similar to soil NO_3_^−^-N, soil urease activity was significantly affected by FTC amplitude, biochar addition rate and FTC frequency (Table 3). Under SFT, soil urease activities of each treatment decreased continuously from the 1st FTC to the 5th FTC, but reversed in the remaining FTCs (Fig. 3a). After the 15th FTC, soil urease activities of BC0, BC2 and BC4 increased by 7.5%, 14.3% and 7.5%, respectively, as compared to their activities after the pre-incubation. Soil urease activities under LFT varied from 21.9 to 30.1 μg NH_4_-N g^−1^ h^−1^ and displayed an initial decrease but subsequently increased with the increase of FTC times. Furthermore, soils amended with biochar always showed higher urease activities than BC0 in both FTC conditions. The mean activities decreased in the following order: BC4 > BC2 > BC0.

In most treatments, soil protease activities decreased as FTCs proceeded (Fig. 3c,d). In comparison with the activities after the pre-incubation, soil protease activities decreased by 17.8–25.1% and 10.5–25.5% after 15 times of FTC under SFT and LFT, respectively, with the largest decrease found in LFT-BC0. Soil protease activity was also significantly affected by biochar addition rates (Table 3), and soil protease activities of BC2 and BC4 were higher than those of BC0 during most of the incubation time. However, neither FTC amplitude nor its interaction effects with other factors significantly influenced protease activities (Table 3).

### N_2_O emissions

**Figure 4:**
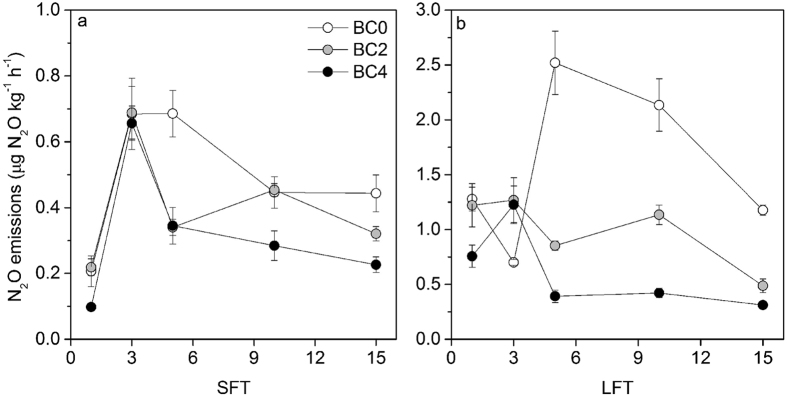
Changes in soil N_2_O emissions by biochar additions during FTC ((**a**), FTC with small amplitude; (**b**), FTC with large amplitude). Bars represent the standard error of the mean (n = 3).

As shown in Table 3, soil N_2_O emission was significantly affected by FTC amplitude, biochar addition rate, FTC frequency, as well as their interaction effects. Under SFT, soil N_2_O emissions of all treatments were low with a range of 0.1 to 0.2 μg N_2_O kg^−1^ h^−1^ after the 1st FTC. Thereafter, sharp increases in N_2_O emissions were observed for all treatments (Fig. 4). The emissions after the 3rd FTC were 3.3, 3.1 and 6.7 times higher than those after the 1st FTC for BC0, BC2 and BC4, respectively. During the rest of FTCs, N_2_O emissions of each treatment showed decreasing tendencies. The emissions of BC0 were generally higher than those of BC2 and BC4. Soil N_2_O emissions of each treatment also showed considerable changes with the increase of FTC times under LFT. For BC2 and BC4, soil N_2_O emissions fluctuated as the incubation continued and the peak emissions were observed after the 3rd FTC. By contrast, soil N_2_O emissions of BC0 first decreased between the 1st FTC and the 3rd FTC, then increased drastically after the 5th FTC, and finally showed a decreasing trend during the last 10 FTCs. Its peak emission was 2.5 μg N_2_O kg^−1^ h^−1^, which was about 2.0 and 2.1 times higher than peak emissions of BC2 and BC4, respectively.

**Figure 5:**
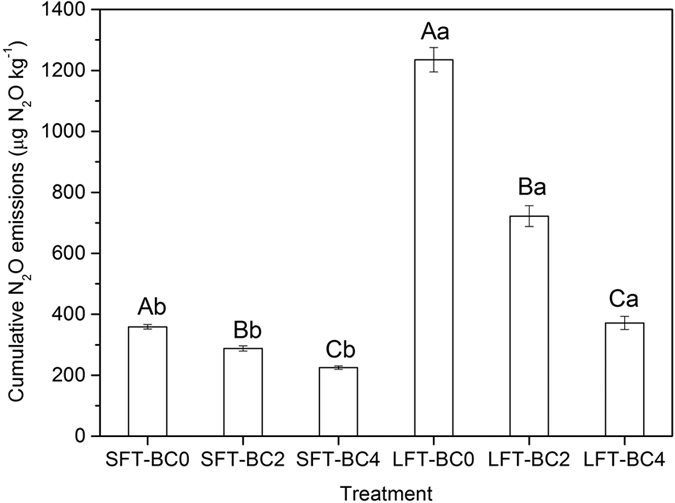
Cumulative N_2_O emissions of each treatment during the whole incubation. Bars represent the standard error of the mean (n = 3). *Uppercase letters* above the bars indicate significant differences among biochar addition rates while under a same FTC amplitude after one-way ANOVA with LSD test (n = 3, *P* < 0.05). *Lowercase letters* above the bars indicate significant differences between FTC amplitudes while under a same biochar addition rate after independent-samples t test (n = 3, *P* < 0.05)

Soil cumulative N_2_O emissions during the whole incubation showed significant differences among different biochar addition rates (Fig. 5). In comparison with BC0, biochar amendments decreased soil N_2_O emissions by 19.9% (BC2) and 37.3% (BC4) under SFT, and by 41.5% (BC2) and 69.9% (BC4) under LFT. Furthermore, LFT induced about 1.7 to 3.4 times higher soil N_2_O emissions than SFT when soils were treated with same biochar addition rates. The highest and lowest cumulative N_2_O emissions were observed in LFT-BC0 and SFT-BC4, respectively.

## Discussion

### Impacts of biochar amendment on soil inorganic N and SMBN contents during FTC

There have been reports of increases in soil NH_4_^+^-N and NO_3_^−^-N contents after treating successive FTCs^33^,^34^. In our study, soil NO_3_^−^-N contents of all treatments also increased during FTC. However, soil NH_4_^+^-N contents of each treatment showed decreasing tendencies, especially within the first five FTCs. Our results suggested that FTC might inhibit the N mineralization, whereas favor the nitrification, which converted NH_4_^+^-N into NO_3_^−^-N under aerobic condition. Besides, previous studies have indicated that the volatilization of soil NH_4_^+^-N can be stimulated under alkaline condition^35^,^36^. Therefore, the decreased soil NH_4_^+^-N contents might be also attributed to the volatilization of NH_4_^+^-N because the soil pH was high in our study (Table 2). Although the effects of biochar on soil N dynamics have been widely investigated, information on how biochar affects soil inorganic N contents during FTC is still limited. Our results showed that biochar amendments significantly decreased soil NO_3_^−^-N content while had little effect on soil NH_4_^+^-N content as compared to BC0 during FTC, suggesting that soil nitrification may be inhibited by adding biochar when FTC occurs. In a laboratory study, Zhang *et al*.^37^ found that both soil NH_4_^+^-N and NO_3_^−^-N contents decreased with adding biochar. They suggested that biochar had the ability to adsorb soil inorganic N, and then led to decreases in soil nitrification and net N mineralization. Christenson *et al*.^38^ observed a significant negative relationship between net nitrification and soil C/N ratio. They suggested that the low gross NH_4_^+^-N production or higher NO_3_^−^-N consumption were the possible reasons for this phenomenon. As shown in Table 2, soil C/N ratio increased from 9.1 to 12.4 or 15.5 by biochar amendments. Therefore, the increased soil C/N ratio might be another possible reason for the decreases in soil nitrification.

Changes in soil microbial biomass can reflect the process of microbial growth, death, and the degradation of soil organic matter^19^. Similar to our results, previous studies have also reported that soil microbial biomass contents could be reduced by FTC^10^,^39^. Such decreases may be attributed to that FTC has a sterilization function, which kills soil microorganisms during freeze periods^40^. Moreover, our results indicated that bicohar amendments had little effects on SMBN contents during FTC. Although the effects of biochar on soil microbial biomass have been extensively investigated, the existing results are still disputable. Most of the related studies reported that soil microbial biomass could be increased by adding biochar^22^,^41^. Some studies demonstrated that biochar is a porous material, which has many pores, especially macropores (>200 nm) on its surface^42^,^43^. These macropores may hold substrates and serve as favorable habitats for soil microorganisms^43^. However, contrary results were also reported. For example, Dempster *et al*.^23^ pointed out that biochar could decrease soil microbial biomass C but not influence SMBN in a pot study. These differences may be explained in part by variations in biochar rate and type (e.g. biochar feedstock, pyrolysis temperature, etc.) along with soil types.

### Impacts of biochar amendment on soil enzyme activities during FTC

Soil enzymes play critical roles in maintaining nutrient availability. Their activities are “sensors” of microbial status and soil physicochemical conditions^18^. In this study, both urease and protease activities decreased during the first five FTCs, suggesting that FTC had a short-term effect on decreasing soil enzyme activities. Previous studies suggested that the decreased enzyme activities during FTC might be attributed to the decreased microbial activities because soil enzymes mainly originate from soil microorganisms^10^,^44^. As an example, Wang *et al*.^10^ reported that soil enzyme activities were significantly correlated to soil microbial biomass C contents during FTC. Similarly, soil enzyme activities also showed significant correlations with SMBN in this study (Table 4), partially supporting the assertion. However, soil enzyme activities were quite stable or even increased after the 5th FTC, suggesting that soil enzymes or microorganisms had probably been adapted to the FTC conditions.

During most of the incubation period, soil urease and protease activities of BC2 and BC4 were generally higher than those of BC0, indicating that biochar might help retaining soil enzyme activities during FTC. This result was in agreement with previous studies which also found that soil enzyme activities were increased by adding biochar^22^,^45^. The potential mechanisms of these increases may be: (1) the macropores of biochar serve as favorable habitats for soil microorganisms and protect them from being killed by FTCs; (2) the increased substrates induced by FTC are fixed on the surface of biochar and can be used by soil microorganisms. However, contrary reports that biochar had no effects or even negative effects on increasing soil enzyme activities also existed^24^,^46^. Hence, more studies of biochar amendment on soil enzyme activities are needed to understand its effect as well as its underlying mechanisms.

### Impacts of biochar amendment on soil N_2_O emissions during FTC

It has been demonstrated that soil N_2_O emissions during freeze-thaw periods are an important part of the annual N_2_O budget. Our results showed that biochar amendment suppressed N_2_O emissions by 19.9–69.9% as compared to BC0 during FTC, suggesting that biochar amendment might be a potential way to mitigate soil N_2_O emissions during FTC. The results of Pearson correlation analysis showed that soil N_2_O emissions were significantly correlated to soil NO_3_^−^-N content (Table 4). As illustrated above, soil nitrification, which converted NH_4_^+^-N to NO_3_^−^-N, might occur during the incubation. In addition, soil NO_3_^−^-N contents of BC2 and BC4 were significantly lower than those of BC0. Therefore, the suppression of N_2_O emissions might be related to the nitrification, which was inhibited by biochar amendments. Some studies indicated that biochar contains volatile organic compounds such as α-pinene and ethylene, which are known as nitrification inhibitors^47^,^48^. Similar to our results, Sarkhot *et al*.^49^ pointed out that biochar amendments led to 68–75% and 26% reductions in net nitrification and N_2_O emission, respectively. They suggested that such reductions were a result of soil inorganic N adsorption. However, Case *et al*.^50^ hypothesized that the decreased soil cumulative N_2_O productions by biochar were related to the biological or physical immobilization of NO_3_^−^-N, which removed large amounts of NO_3_^−^-N from the extractable pool. Therefore, the adsorption of soil inorganic N as well as the ^15^N tracer experiments are suggested to be designed in the future to have a better understanding of the mechanisms of N_2_O suppression by biochar amendment during FTC.

## Conclusion

Results of the present study showed that soil N_2_O emissions from a cultivated sandy loam soil could be suppressed by adding biochar during FTC. The decreased nitrification indicated by the lower soil NO_3_^−^-N contents in the biochar treatments was found to play an important role in such suppressions. Biochar amendments also had a positive effect on retaining soil urease and protease activities, while it did not affect NH_4_^+^-N and SMBN contents during FTC. However, soil NH_4_^+^-N and SMBN contents, urease and protease activities did not show significant correlations to soil N_2_O emissions. Our study indicates that biochar amendment can be a potential method to reduce soil N_2_O emissions during freeze-thaw periods. Although extrapolation of the findings from this short-term laboratory study to long-term field results should be conducted with caution, the results still gave an insight into how biochar affects soil N_2_O emissions during FTC. Further studies are needed to estimate the effectiveness of biochar amendment on reducing freeze-thaw induced N_2_O emissions from different soils under field conditions.

## Additional Information

**How to cite this article**: Liu, X. *et al*. Response of N_2_O emissions to biochar amendment in a cultivated sandy loam soil during freeze-thaw cycles. *Sci. Rep.* **6**, 35411; doi: 10.1038/srep35411 (2016).

## Figures and Tables

**Table 1:** The physicochemical properties of soil and biochar (mean ± SE^a^, n = 3).

Properties	Soil	Biochar
Organic C (g C kg^−1^)	11.0 ± 0.2	—b
TN (g N kg^−1^/ %_db_^c^)^d^	1.2 ± 0.1	1.0 ± 0.1
NH_4_^+^-N (mg N kg^−1^)	4.0 ± 0.10	3.1 ± 0.2
NO_3_^−^-N (mg N kg^−1^)	21.5 ± 0.1	1.8 ± 0.03
pH _1:5_ (H_2_O)	8.0 ± 0.04	9.2 ± 0.1
Electric conductivity_1:5_ (μs cm^−1^)	315.3 ± 2.1	2393.3 ± 57.7
Ash content (%_db_)	—	22.7 ± 0.9
Total C (%_db_)	—	66.6 ± 3.7
Total H (%_db_)	—	2.2 ± 0.2
Total S (%_db_)	—	0.4 ± 0.04
Total O (%_db_)	—	11.1 ± 2.1

^a^standard error.

^b^not detected.

^c^dry basis.

^d^“g N kg^−1^” for soil and “%_db_” for biochar.

**Table 2:** Treatment layout and physicochemical properties of the mixtures of soil and biochar after pre-incubations (mean ± SE^a^, n = 3).

Biochar rate (w/w %)	FTC treatment	Code	Properties of mixture				
			pH _1:5_ (H_2_O)	Electric conductivity_1:5_ (μs cm^−1^)	SOC (g C kg^−1^)	TN (g N kg^−1^)	C/N
0	−5 ~ 5 °C	SFT^b^-BC0	7.9 ± 0.1	337.7 ± 6.8	11.0 ± 0.2	1.2 ± 0.1	9.1 ± 0.8
0	−10 ~ 10 °C	SFT-BC0					
2	−5 ~ 5 °C	SFT-BC2	8.0 ± 0.01	355.7 ± 5.7	19.5 ± 0.2	1.6 ± 0.01	12.4 ± 0.1
2	−10 ~ 10 °C	LFT-BC2					
4	−5 ~ 5 °C	LFT-BC4	8.0 ± 0.01	385.3 ± 3.0	29.2 ± 0.5	1.9 ± 0.04	15.5 ± 0.2
4	−10 ~ 10 °C	LFT-BC4					

^a^SE, standard error.

^b^SFT, FTC with small amplitude.

^c^LFT, FTC with large amplitude.

**Table 3:** Results of three-way ANOVAs (*F* values) testing the effects of FTC amplitudes, biochar addition rates and FTC frequencies on soil NO_3_^−^-N, NH_4_^+^-N, SMBN, urease activities, protease activities and N_2_O emissions.

Source	NO_3_^−^-N	NH_4_^+^-N	SMBN	Urease	Protease	N_2_O
FTC amplitude	5.967*	0.197	61.365**	29.552**	0.317	55.874**
Biochar addition rate	38.313**	2.158	0.253	8.093**	8.097**	12.258**
FTC frequency	19.447**	81.313**	4.523*	12.602**	6.677**	3.234*
FTC amplitude×Biochar addtion rate	3.068	3.596*	3.918*	0.166	1.250	20.003**
FTC amplitude×FTC frequency	3.861*	3.566*	2.537*	4.961*	1.440	4.701*
Biochar addition rate×FTC frequency	4.427**	3.183*	2.515*	1.043	1.378	12.055**
FTC amplitude×Biochar addition rate×FTC frequency	3.889**	1.884	0.975	1.339	0.682	8.048**

Boldface values indicate effects were significant. *P < 0.05; **P < 0.01.

**Table 4:** Pearson coefficients (R) of the correlations among soil NO_3_^−^-N contents, NH_4_^+^-N contents, SMBN contents, ucrease activities, protease activities and N_2_O emissions.

	NO_3_^−^-N	NH_4_^+^-N	SMBN	Urease	Protease
NO_3_^−^-N	1				
NH_4_^+^-N	−0.273**	1			
SMBN	−0.131	0.215*	1		
Urease	−0.196	0.400**	0.431**	1	
Protease	−0.221*	0.516**	0.344**	0.490**	1
N_2_O	0.360**	−0.066	−0.114	−0.385**	−0.077

Boldface values indicate effects were significant. *P < 0.05; **P < 0.01.
